# Bibliometric analysis of COVID‐19 publications shows the importance of telemedicine and equitable access to the internet during the pandemic and beyond

**DOI:** 10.1111/hir.12465

**Published:** 2022-11-13

**Authors:** Mahnaz Samadbeik, Peivand Bastani, Farhad Fatehi

**Affiliations:** ^1^ Social Determinants of Health Research Center Lorestan University of Medical Sciences Khorramabad Iran; ^2^ School of Allied Medical Sciences Lorestan University of Medical Sciences Khorramabad Iran; ^3^ School of Dentistry, UQ Oral Health Centre The University of Queensland Brisbane Australia; ^4^ School of Psychological Sciences Monash University Melbourne Australia; ^5^ Centre for Health Services Research The University of Queensland Brisbane Australia

**Keywords:** digital divide, health literacy, information literacy, information need, internet access, pandemic, telemedicine

## Abstract

**Background:**

Pandemics highlight the increasing role of information and communications technology for improving access to health care. This study aimed to present a bibliometric analysis of the concept of digital divide reported in the published articles concerning the coronavirus disease 2019 (COVID‐19) pandemic.

**Methods:**

To conduct this bibliometric analysis of research topics and trends, we used VOSviewer software. We developed a search strategy to retrieve peer‐reviewed publications related to ‘digital divide in the COVID‐19 era' from the Scopus database.

**Results:**

In total, 241 publications on the topic of digital divide and COVID‐19 were retrieved from Scopus database between 2020 and 2021. The analysis of keywords co‐occurrence of research topics revealed four main clusters including: ‘telemedicine’, ‘Internet access and Internet use’, ‘e‐learning’ and ‘epidemiology’. Seven characteristic categories were examined in these research topics, including: sociodemographic, economic, social, cultural, personal, material and motivational.

**Conclusion:**

‘Telemedicine’ and ‘Internet access and Internet use’ as the largest clusters are connected to topics addressing inequalities in online health care access. Thus, policymakers should develop or modify policies in more egalitarian Internet access for all community members not only during a pandemic like the COVID‐19 but also at regular times.

**Key messages**

- Policymakers should develop or modify policies for more egalitarian Internet access for all community members, especially during a pandemic like the COVID‐19.
- Increasing access to the Internet should be accompanied by digital skills support, so that people with low health literacy can be helped to make the most of digital health services and telemedicine.

## BACKGROUND

COVID‐19 emerged as a severe contagious respiratory disease after severe acute respiratory syndrome (SARS) and middle East respiratory syndrome (MERS) at the last days of 2019 and rapidly spread around the world. The severity and the spread of the disease in several continents made the World Health Organization announce it as a new pandemic in March 2021 (Rothan & Byrareddy, 2020). It is obvious that such an emergency condition with its unknown nature caused a wave of fear and panic worldwide particularly in the countries with higher rates of morbidity and mortality (Li et al., 2020). Different mental and psychological effects such as anxiety, depression and stress are also reported as the negative consequences of the outbreak along with physical disorders and death (Turna et al., 2021).

In a public health crisis such as the COVID‐19 pandemic, access to accurate and timeliness information can help the communities behave more consciously and protectively. At the same time, pandemic circumstances may cause more interests and tendencies for utilizing telehealth services, telemonitoring and other technologies to facilitate the provision of health services and the exchange of health information. These types of information and services can be shared easily via social media, mobile apps, telehealth and new services for epidemiological monitoring (Gabbiadini et al., 2020). These technologies can facilitate access to the health services in order to minimize the risk of spread of the pandemic and to improve the utilization of health services in the pandemic restrictions such as quarantine.

Digital divide as a specialized concept is defined as any sort of inequality to the access and utilization of the modern information and communication technology (ICT), including the Internet (Castells, 2002). This inequality can be seen in three levels. The first level is the Internet access divide; the second level is digital skills; and finally, the third level is the outcome (of utilizing the Internet). According to the literature, these levels of digital divide can be caused by different socio‐economical, cultural, personal and motivational determinants. Scientific evidence indicates a number of sub‐determinants, including household income (Scheerder et al., 2017), age (Giansanti & Velcro, 2021), level of education and literacy and type of skills (Jaana & Paré, 2020).

According to recent studies, pandemics can lead to a particular condition that highlights the importance of access to the Internet and credible health information to increase the utilization of the health services for different population groups. In this regard, the governments have different approaches to facilitate access to the Internet and ICTs for their citizens. For instance, attempts were made to increase the access to educational resources through e‐learning for students and their educators (Correia, 2020), developing telehealth for screening and providing health care services, online tracking mobile applications, tele‐ICUs and remote consultation (Sageena et al., 2021).

The concept of inequality caused by digital divide is highlighted differently by various authors. For instance, although some evidence has emphasized the risk of excluding some members of the society, particularly those who live in remote and rural areas, from access to digital technologies for diagnosis, follow‐up and treatment (Bryant et al., 2020), other evidence has focused on the opportunities that telehealth can provide for the residents of rural areas. At the same time, telehealth and digital health can decrease exposure to COVID hot spots, and thus reduce likelihood of contamination and infection during the pandemic (Sageena et al., 2021). It would be obvious that this area needs evidence‐informed interventions by the governments in order to decrease the gaps, increase the access to the health services and move towards equality in health care.

Despite this extensive area of influence of technologies during pandemics, the general picture of research on the contexts and contents of this topic remains unknown. Undoubtedly, it can be considered as an opportunity for health policymakers and health care managers to become more familiar with the concept of the digital divide and its characteristics. Such a new knowledge can pave the way for health policymakers for better identification of the stakeholders, more realistic planning and interventions to improve access to the relevant technology, information and telehealth. Thus, this study aimed to present a bibliometric analysis of the concept of digital divide reported in published articles in the COVID‐19 pandemic era during 2020 and 2021.

## METHODS

To conduct this bibliometric analysis of research topics and trends, we used VOSviewer software version 1.6.15 (Van Eck & Waltman, 2010). We developed a search strategy to search Scopus database and retrieve the records of peer‐reviewed publications related to ‘digital divide’ and ‘COVID‐19’. Table 1 shows our search strategy for the Scopus database. The Scopus database was selected for this study because it covers more titles and research papers than other popular biomedical databases (Leydesdorff et al., 2010).

**TABLE 1:** Search strategy for Scopus database

No	Search query	Search field/limits
#1	‘Internet availability’ OR ‘Internet access’ OR ‘access of Internet’ OR ‘availability to Internet’ OR ‘availability of Internet’	In: Topic (Title, Abstract, Keywords)
#2	‘Internet skills’ OR ‘digital skills OR ‘online skills’	
#3	‘Internet use’ OR ‘Internet activities’ OR ‘online activities’ OR ‘Internet usage’	
#4	‘effects of Internet’ OR ‘Internet effects’ OR ‘outcomes of Internet’ OR ‘Internet outcomes’ OR ‘benefits of Internet’ OR ‘Internet benefits’ OR ‘Internet opportunities’	
#5	indicators OR predictors OR determinants	
#6	(#1 OR #2 OR #3 OR #4) AND #5	
#7	‘digital divide’ OR ‘digital gap’ OR ‘digital health inequity’ OR ‘digital inequality’ OR ‘virtual inequality’	
#8	#6 OR #7	
#9	coronavirus OR COVID‐19 OR ‘severe acute respiratory coronavirus 2’ OR SARS‐coronavirus 2 (CoV‐2) OR ‘2019‐nCoV’ OR nCoV OR ‘2019 novel coronavirus’ OR ‘novel coronavirus’ OR ‘acute respiratory syndrome coronavirus 2’ OR SARS‐CoV‐2	
#10	#8 AND #9	Language: English Timespan: 2020–2021

The Scopus search result was exported in the format of a CSV file with all data elements, including information on citation, bibliography, abstract and keywords. The electronic search was carried out in May 2021.

To construct a network, the Scopus results file was imported into VOSviewer software. Using this software, we visualized all keywords (author keywords and index keywords) related to ‘digital divide’ and ‘COVID‐19’ via co‐occurrence analysis with a full counting method. In order to simplify the maps and reduce term density, we set the threshold of minimum number of occurrences of a keyword to 5. So, terms with fewer than five occurrences were not shown on the map. This threshold is a desirable number to cancel misspelled keywords as well as nugatory ones. Keywords network analysis was performed to display the most researched keywords in this topic and their relationship. We also used VOSviewer to demonstrate international collaboration through co‐authorship analysis.

Two types of maps were created for visualizing keywords associations: network visualization and density visualization. In network visualization, items are demonstrated by nodes and labels, and classified in clusters. The size of the node and the label of an item is determined by the weight of the item. The larger the weight of an item, the bigger the label and the circle denoting the item. The weight of an item is determined by the links and the total link strength attributes. Links indicate the number of links of an item with other items and the total link strength represents the overall strength of the links of an item with other items (Van Eck & Waltman, 2019).

Clustering is a method to set items into groups by similarity and detect closely associated items (van Eck & Waltman, 2014). In these maps, clusters of items are presented by different colours to represent the cluster to which a node has been allocated. The clustering technique that was used in this study is explained in detail by Waltman et al. (2010).

The item density visualization helps detect dense and important areas in the map. The more frequency of occurrence of the item, the denser the area. In this visualization, attention has been paid to the colour and distribution of each point. Each point has a colour depending on the density of items at that point. The range of colour is from blue to green to yellow. The yellow colour indicates the highest number of items in the neighbourhood of the item and the highest weights of the neighbouring items density, followed by green and then blue. Conversely, the smaller the number of items in the neighbourhood of the item and the lower the weights of the neighbouring items density, the closer the colour of the point is to blue (van Eck & Waltman, 2014; Wang et al., 2021).

For data cleaning and terms merging, a thesaurus was created to enable a meaningful concept mapping. For this purpose, key words were first inspected with the aims of converting all singular terms into plural ones (e.g., from ‘health care disparity’ to ‘health care disparities’) and, ignoring the terms denoting the main concepts of the study and general terms that did not add value to the study (e.g., ‘coronaviruses’, ‘digital divide’, ‘human’, ‘cross‐sectional study’, etc.). After this step, the thesaurus file was generated by entering these key words as directed by the instruction manual of VOSviewer into a simple text editor (Notepad++ 7.9.2 for Windows) and, then this thesaurus file was uploaded into VOSviewer to replace and ignore the specified terms for creating maps.

For the purpose of this study, three levels of digital divide were considered from a study by Scheerder et al. (2017), These three levels are: (1) Internet access (first‐level digital divide), (2) Internet skills and use (second‐level digital divide) and (3) Internet use (third‐level digital divide).

## RESULTS

### Bibliometric analysis of publication output

In total, 241 publications on the topic of digital divide and COVID‐19 were retrieved from the Scopus database from January 2020 to the end of May 2021, which included 173 (65.8%) original research articles, 27 (10.3%) review articles, 27 (10.3%) conference papers, 17 (6.4%) notes, 10 (3.8%) editorials and 9 (3.4%) letters. Of these documents, 128 (53.1%) papers were published in 2020 and the other 113 (46%) were published in 2021.

### Country distribution and collaborations on research related to the COVID‐19 digital divide

The top countries in terms of publications related to the COVID‐19 and digital divide were United States (*n* = 87, 36.1%), United Kingdom (*n* = 25, 10.4%), Canada (*n* = 17, 17.1%), India (*n* = 17, 17.1%), Italy (*n* = 16, 6.6%), Spain (*n* = 14, 5.8%), Australia (*n* = 12, 5%), The Netherlands (*n* = 8, 3.3%), South Africa (*n* = 8, 3.3%), Indonesia (*n* = 7, 2.9%) and Switzerland (*n* = 7, 2.9%). About 50% of publications were published in the United States and the United Kingdom (Table 2).

**TABLE 2:** Top countries in terms of number of publications related to digital divide and COVID‐19

Country	Number of publications
United States	87
United Kingdom	26
Canada	17
India	17
Italy	16
Spain	14
Australia	12
The Netherlands	8
South Africa	8
Indonesia	7

**FIGURE 1:**
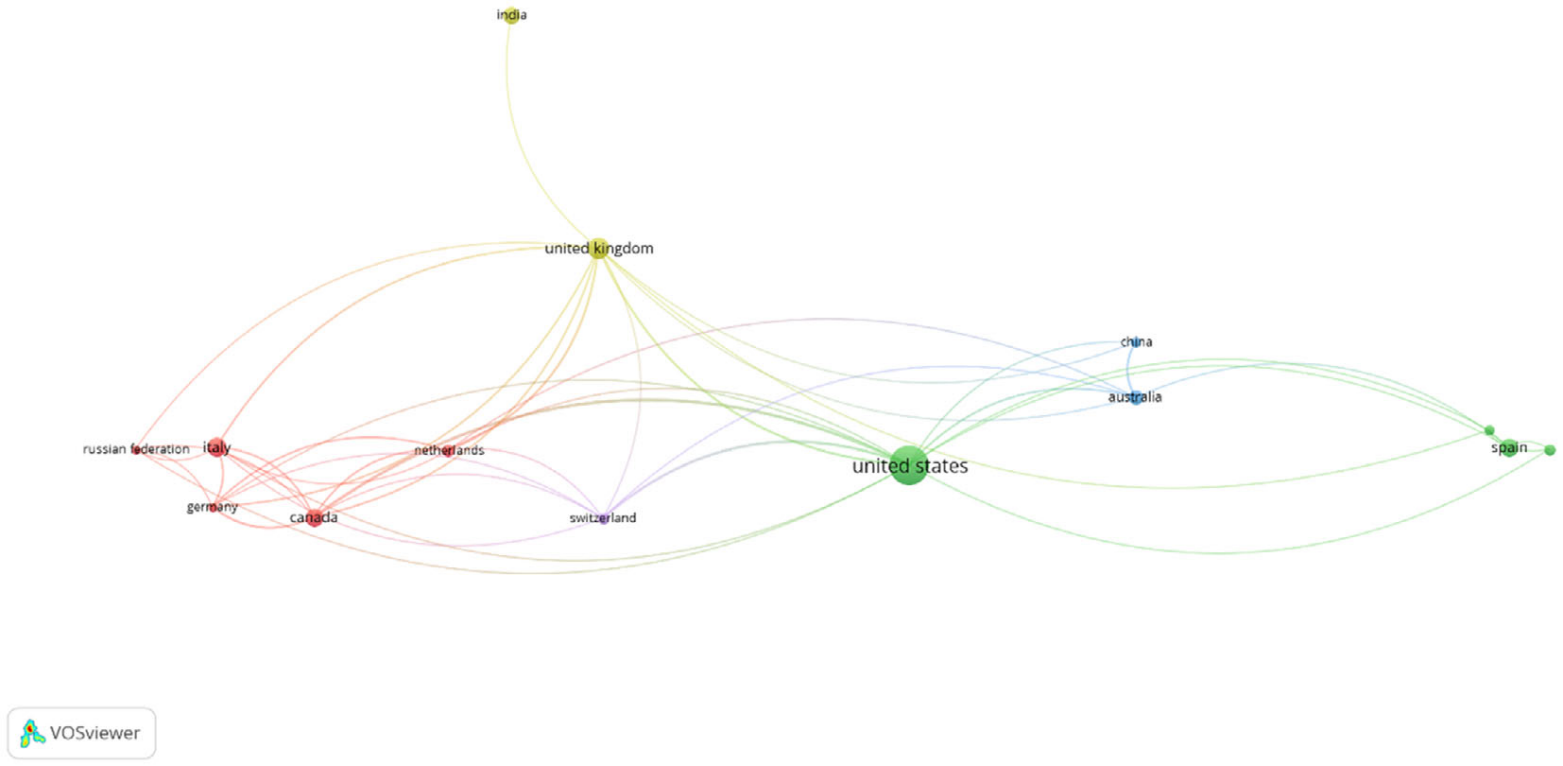
Network visualization map of collaboration between the countries. Each node indicates a country and the lines between the nodes represent the strength of relation between countries. The size of the nodes is proportional to the number of documents from each country. The closer the nodes to each other, the stronger level of collaboration in the publication of the papers; and the thicker the line connecting two countries, the number of shared publications.

The co‐authorship map of countries (Figure 1) depicts the countries by circles and co‐authorship between them by links. The threshold of 5 was used for creating the co‐authorship country map. The thickness of the links and the distance between the nodes shows the collaboration strength between the countries. The map consisted of five clusters shown by different colours as follows: three countries surrounding the United States (green), one country adjacent to the United Kingdom (yellow), one country next to Australia (blue) and only Switzerland in cluster 5 (purple). The United States and United Kingdom had the strongest collaboration with other countries with 22 and 18 collaborators, respectively. It indicates that geographical proximity is not a determinant factor in scientific collaboration.

### Bibliometric analysis of the keywords

**FIGURE 2:**
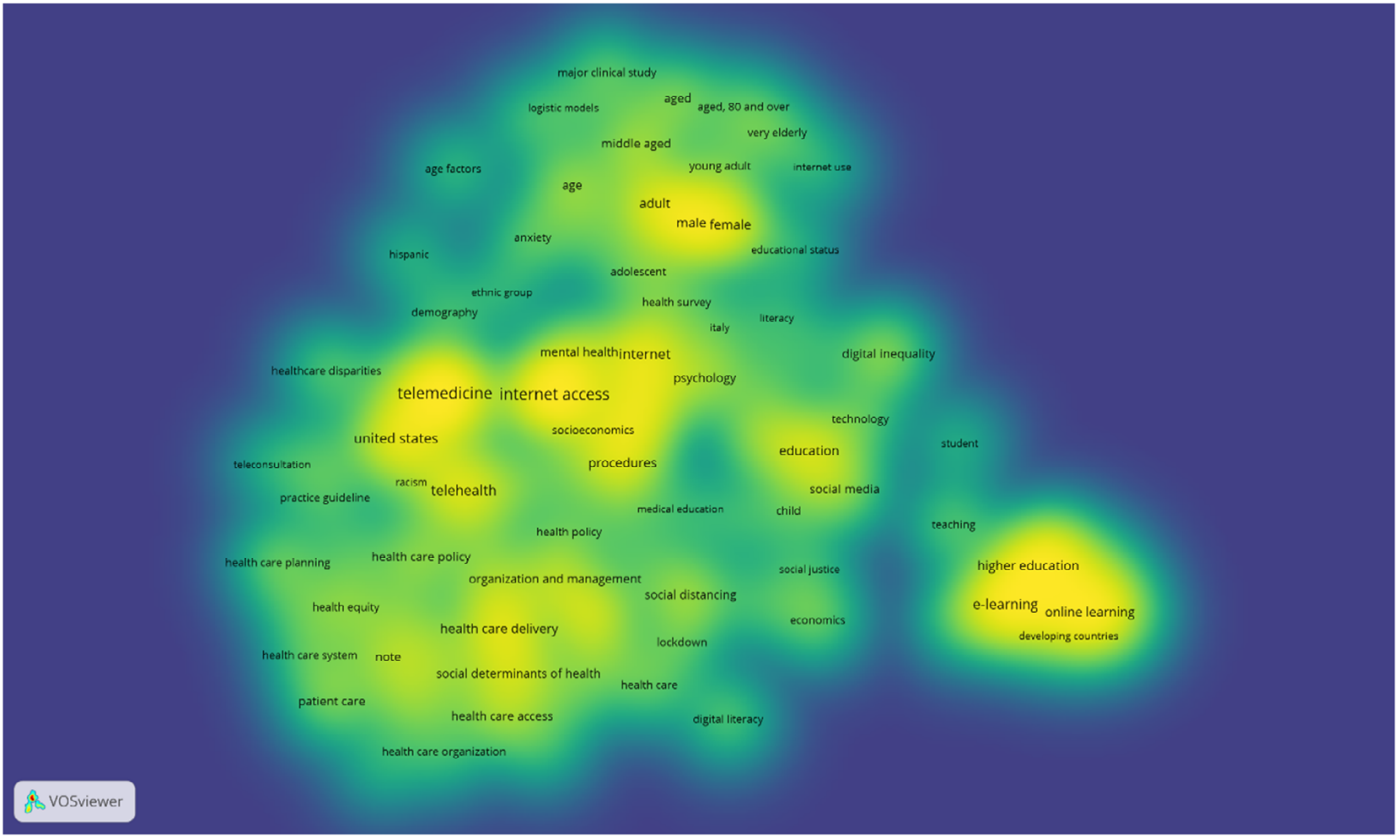
Item density visualization of keywords co‐occurrences. Areas with higher density represent the keywords with more citations. Each point has a colour indicating the density of keywords at that point. Colours range from blue (low density) to green (medium density) and yellow (high density).

To visualize the most frequent terms used for the research topics, two maps were created: keywords network map and item density map. Figure 2 shows the keywords density map generated by VOSviewer. The largest numbers and the highest weight of neighbouring keywords were attributed to ‘internet access’, ‘telemedicine’, ‘higher education’, ‘telehealth’, ‘female’ and ‘male’, depicted by yellow colour. The smallest number and the lowest weights of the neighbouring keywords were attributed to ‘health care disparities’, ‘health care organization’, ‘digital literacy’, ‘teleconsultation’ and ‘age factors’, as is shown by blue colour.

Network visualization map indicating co‐occurrence of all keywords of the paper occurred for more than five times. The total link strength indicates the total strength of the co‐occurrence links of a given keyword with other keywords. Of the 1753 keywords, 102 met or exceeded the threshold of five occurrences and appeared on the map. The keywords that appeared most were ‘Internet access’ (total link strength 311) and ‘telemedicine’ (total link strength 271).

**FIGURE 3:**
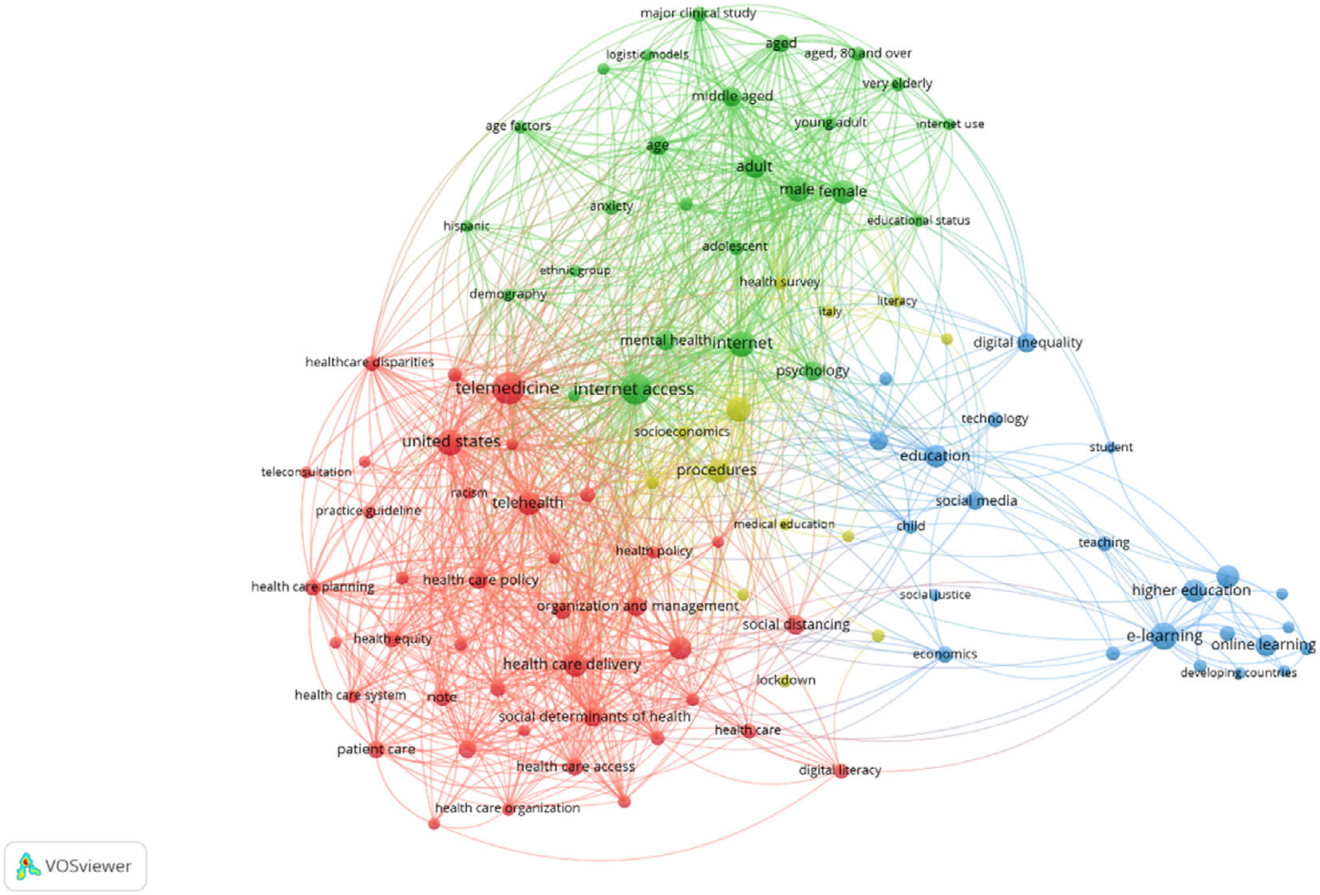
Network visualization of keywords co‐occurrences. Each node shows a keyword; the links indicate the association between keywords; the colour and distance between nodes represent the similarity between keywords. The size of a node shows the number of publications in which the term was found, and the distance between two terms indicates the relatedness of the terms.

The analysis of keywords co‐occurrence of research topics revealed four clusters depicted in Figure 3 with different colours. Colours indicate groups of keywords that are fairly strongly related to each other. These groups were detected using the clustering technique of VOSviewer. This map shows different groups of keywords covered by clusters, including: (1) ‘social determinants and ‘telemedicine’; (2) ‘Internet access’ and ‘Internet use’, and characteristics examined in relation to these levels; social determinants of health and telemedicine; (3) ‘digital inequality’ and ‘eLearning’; and (4) ‘epidemiology’.

The clusters and nodes of research topics are summarized in Table 3, and include the followings:

**TABLE 3:** Digital divide research concepts and hot topics

Cluster	Concept	Nodes (*n* = 102)
**1**	Telemedicine	Access to information, African American, aging, digital literacy, financial management, health care, health care access, health care cost, health care delivery, health care organization, health care personnel, health care planning, health care policy, health care system, health disparities, health equity, health literacy, health policy, health services accessibility, health care disparities, medical information, mental disease, note, organization and management, patient care, patient safety, poverty, practice guideline, public health, racism, rural population, severe acute respiratory syndrome coronavirus, social determinants of health, social distancing, social status, teleconsultation, telehealth, telemedicine, United states, videoconferencing (*n* = 42)
**2**	Internet access and Internet use	Adolescent, adult, age, age factor, aged, aged 80 and over, anxiety, demography, educational status, ethnic group, female, Hispanic, Internet, Internet access, Internet use, logistic models, major clinical study, male, mental health, middle aged, minority group, psychology, statistical model, surveys and questionnaires, very elderly, young adult (*n* = 26)
**3**	e‐learning	Child, developing countries, digital inequality, digital skills, digital technologies, e‐learning, economics, education, educational institutions, higher education, learning systems, online learning, social isolation, social justice, social media, social support, student, students, surveys, teaching, teaching and learning, technology (*n* = 23)
**4**	Epidemiology	Digital technology, epidemic, epidemiology, ethnology, health survey, Italy, literacy, lockdown, medical education, procedures, psychological well‐being, socioeconomics, United States (*n* = 13)

#### Cluster 1: Telemedicine

This cluster is the largest cluster in our study (40 items). ‘Telemedicine’ has the largest occurrence in selected keywords (occurrence = 33, total link strength = 271), and is closely connected to topics concerning ‘health care disparities’, ‘social determinants’, ‘Internet access’, ‘digital literacy’ and ‘health policy’.

#### Cluster 2: ‘Internet access’ and ‘internet use’

The second large cluster is composed of 26 keywords. Internet access has the highest total link strength across the article's topics (occurrence = 32, total link strength = 311). In this cluster, Internet access and Internet use are related to ‘age factor’, ‘demography’, ‘educational status’, ‘ethnic group’ and ‘socioeconomics’ in the publications.

#### Cluster 3: e‐learning

The third cluster is composed of 23 items. The areas of research in e‐learning have connections with ‘online learning’, ‘e‐learning’, ‘online learning’, ‘higher education’, ‘teaching’ and ‘developing countries’.

#### Cluster 4: Epidemiology

The Epidemiology cluster is relatively smaller than the previous three clusters (13 items). The ‘epidemiology’ has close connections to ‘socioeconomics’, ‘ethnology’, ‘health survey’ and ‘epidemic’.

### Digital divide levels

The analysis of research topics in this area shows that Internet access (first‐level digital divide) and Internet use (second‐level digital divide) were noted in publications about the COVID‐19. These topics are mainly represented by Cluster 2.

### Characteristics examined in relation to digital divide

Characteristics examined in this research area were extracted from all clusters' keywords. Categorization of these characteristics was based on Scheerder's article (Gabbiadini et al., 2020), and established seven determinant categories, including: sociodemographic, economic, social, cultural, personal, material and motivational. All seven characteristics categories were considered in research topics of digital divide and COVID‐19 pandemic (Table 4).

**TABLE 4:** Characteristics examined in relation to digital divide

Category	Characteristics	Cluster
Sociodemographic	Aging	Cluster 1
	Age	Cluster 2
	Demography	Cluster 2
	Rural population	Cluster 1
Social	Social determinants of health	Cluster 1
	Socioeconomics	Cluster 3
	Social status	Cluster 1
	Social isolation	Cluster 3
	Social support	Cluster 3
Motivational	Digital literacy	Cluster 1
	Health care policy	Cluster 1
	Health policy	Cluster 1
Cultural	Racism	Cluster 1
	ethnic group	Cluster 2
Personal	Literacy	Cluster 3
	Mental disease	Cluster 1
	Mental health	Cluster 2
Economic	Financial	Cluster 2
	Economics	Cluster 3
	Poverty	Cluster 1
	Socioeconomics	Cluster 3
	Educational	Cluster 1
	Education	Cluster 3
Digital skills	Digital skills	Cluster 3

## DISCUSSION

In this study, we visualized the research output concerning digital divide and COVID‐19. Bibliometric and network analysis of the keywords of 241 articles published on this topic revealed four main clusters including: ‘telemedicine’, ‘Internet access and Internet use’, ‘e‐learning’ and ‘epidemiology’.

Telemedicine, which is the largest cluster, is connected to topics addressing inequalities and disparities in health care access. Therefore, it seems that telemedicine access has been studied with respect to topics on determinants of digital divides, such as ‘social determinants’, ‘Internet access’, ‘digital literacy’ and ‘health policy’. On the other hand, it seems that development of telehealth solutions has the potential to help decrease inequality in health care access by all the community members, particularly those who live in rural and remote areas and during specific circumstances such as the COVID‐19 pandemic. In this regard, Sageena et al. has claimed that telehealth can be considered as a new solution for the Indian community to access health care services during pandemics (Gabbiadini et al., 2020). Furthermore, the potential application of telehealth care is emphasized for improving access to health care services in rural and remote places (Mistry, 2012). However, disparities in access to the Internet may affect the equitable access to telehealth options (Jain et al., 2021; Sieck et al., 2021). In this regard, several studies have raised concerns about how the digital divide can increase inequalities in Internet access and digital literacy (Andreou & Svoli, 2013; Fuchs, 2009; Scheerder et al., 2017). Thus, procuring the facilities for access and utilization of the telehealth care services can be among the main concerns of the policymakers in some countries with poor infrastructures of ICTs and Internet. Scott and Mars (2015) have reported that the main challenge in the development of telehealth care services among under‐developed and developing countries is the lack of access to the Internet, as well as digital skills of the health care workers that should be considered in policy making (Scott & Mars, 2015). The COVID‐19 pandemic highlights current disparities in access to health care. Since substituting in‐person visits with telehealth can decrease the risk of exposure to coronavirus, practices should be applied for screening people based on their digital literacy and to provide them with the required training regarding telehealth (Sieck et al., 2021). These measures can decrease inequalities in access to and use of telehealth and enable providers and patients to take advantage of the potential benefits of telehealth. Moreover, policymakers should promote or change policies to more equal Internet access for public not only during a pandemic crisis similar to the COVID‐19 but also in normal times.

The second cluster of research topics, Internet access and Internet use, was related to sociodemographic and economic characteristics, such as ‘age’, ‘demography’, ‘educational status’, ‘ethnic group’ and ‘socioeconomic status’. Previous studies about digital divide also highlighted that the sociodemographic and socioeconomic characteristics were the most common determinants identified in both the second and third levels of digital divide across the studies (Bartikowski et al., 2018; Kim et al., 2013; Kontos et al., 2010). In addition, these characteristics are associated with Internet access and use for health purposes (Andreou & Svoli, 2013; Estacio et al., 2019; Scheerder et al., 2017). Internet access and use were distributed differently between genders, ages, ethnic groups, educational levels and economic levels (Blank & Groselj, 2014; Helsper, 2010; Scheerder et al., 2017). However, some studies showed no relationship between the demographic characteristics the same as age, gender, income and education level and the Internet access (Ahmed et al., 2015). Therefore, in addition to increasing access to the Internet, digital skills development should be employed to lead towards digitization of health care, especially among populations with low digital literacy.

The other results of the study have focused on the connection between e‐learning and concepts related to online and virtual learning environments. E‐learning is emphasized, and is highlighted using the Internet and ICT. During the COVID‐19 pandemic, the application of e‐learning has increased among students in all levels, from elementary schools to colleges and universities. It has become obvious that the degree of acceptance and satisfaction of the online or off‐line users greatly depend on the feasibility of the virtual facilities, Internet access, trusted digital technologies, proper digital skills and appropriate regulations developed by the policymakers (Mahyoob, 2020). Policymakers should be also more sensitive to developing strategic plans for succeeding in the implementation and development of modern technologies of e‐learning as a change paradigm during and after this pandemic (Zalat et al., 2021). Also, topics related to social support (social support, social justice, social media and social isolation) were connected to the topics concerning e‐learning among the published articles. Therefore, social support by peers or by the Internet community is a key to narrowing the learning divide especially in people with different socio‐demographic backgrounds (Vandenbroeck et al., 2008).

Finally, the epidemiology cluster has close connections to ‘socioeconomics’, ‘ethnology’, ‘literacy’ and ‘psychological well‐being’. Previous studies have also shown that the Internet access is unequal among individuals with different ethnicity and psychological well‐being categories (Bartikowski et al., 2018; Salmi, 2020). The role of preparing electronic health data as an input for evidence‐informed policymaking in the scope of epidemiology of the diseases along with the aetiology and cause‐and‐effect cycles of the diseases should not be neglected particularly during pandemics as a prerequisite of managing the outbreaks. This kind of policy making will improve the surveillance system and readiness of the health systems to respond to the new waves of the current COVID‐19 pandemic or future public health disasters (Mohammadpour et al., 2021).

## CONCLUSIONS

‘Telemedicine’ and ‘Internet access and Internet use’ appear to be the most researched topics in digital health for the COVID‐19, addressing inequalities in health care access, sociodemographic and economic characteristics. Thus, policymakers should develop or modify policies in more egalitarian Internet access for all community members not only during a pandemic but also at normal times. In addition to increasing and widening access to the Internet, digital skills development should be employed to lead towards digitization of health care, especially among low digital literacy groups.

## AUTHORS' CONTRIBUTIONS

Mahnaz Samadbeik designed the study, assisted in the data analysis and interpretation and prepared the initial draft of the article. Peivand Bastani contributed to data collection and analysis, and revised the article. Farhad Fatehi contributed to the design of study, assisted the interpretation of the findings and revised the article. All the authors read and approved the final article.

## CONFLICT OF INTEREST

The authors have no conflict of interest to disclose.

## ETHICS STATEMENT

This study was exempted from ethical review as there no participants involved in this study.

## CONSENT FOR PUBLICATION

Not applicable.

## Data Availability

The data that support the findings of this study are available from the corresponding author upon reasonable request.
